# Cognitive modulation of local and callosal neural interactions in decision making

**DOI:** 10.3389/fnins.2014.00245

**Published:** 2014-08-11

**Authors:** Hugo Merchant, David A. Crowe, Antonio F. Fortes, Apostolos P. Georgopoulos

**Affiliations:** ^1^Department of Behavioral and Cognitive Neurobiology, Instituto de Neurobiología, UNAMQuerétaro, México; ^2^Biology Department, Augsburg CollegeMinneapolis, MN, USA; ^3^Department of Veterans Affairs, Minneapolis Health Care System, Brain Sciences CenterMinneapolis, MN, USA; ^4^Department of Neuroscience, University of Minnesota Medical SchoolMinneapolis, MN, USA

**Keywords:** parietal cortex, spatial cognition, synchronicity, monkey

## Abstract

Traditionally, the neurophysiological mechanisms of cognitive processing have been investigated at the single cell level. Here we show that the dynamic, millisecond-by-millisecond, interactions between neuronal events measured by local field potentials are modulated in an orderly fashion by key task variables of a space categorization task performed by monkeys. These interactions were stronger during periods of higher cognitive load and varied in sign (positive, negative). They were observed both within area 7a of the posterior parietal cortex and between symmetric 7a areas of the two hemispheres. Time lags for maximum interactions were longer for opposite- vs. same-hemisphere recordings, and lags for negative interactions were longer than for positive interactions in both recording sites. These findings underscore the involvement of dynamic neuronal interactions in cognitive processing within and across hemispheres. They also provide accurate estimates of lags in callosal interactions, very comparable to similar estimates of callosal conduction delays derived from neuroanatomical measurements (Caminiti et al., 2013).

## Introduction

During different stages of a demanding task, synaptic interactions among neurons may change, depending on the network(s) to which they belong and the level of processing required. Indeed, the computations performed by a neural network could be better understood by investigating (a) how constituent neurons are interacting in a given time window, and (b) how these interactions change from epoch to epoch. In a categorization task, for example, the initial epoch consists in the presentation of a single stimulus that subsequently is mentally assigned (through a decision process) to a group or category whose members are treated equally; and finally, a particular movement is executed to express the result of the decision. Many studies have characterized the response properties of neurons in diverse brain areas during the categorization of different features of visual (Freedman et al., 2001, 2002; Merchant et al., 2001, 2011; Freedman and Assad, 2006; Crowe et al., 2013) and tactile stimuli (Romo et al., 1993, 1997; Merchant et al., 1997). Nevertheless, little is known about the dynamic interactions inside a categorization circuit. These interactions can be determined using action potentials or local field potentials (LFPs), which are neural signals recorded by extracellular electrodes in behaving animals. Action potentials last ~1 ms and are emitted by cells in spike trains, whereas LFPs are complex signals determined by the input activity of an area in terms of population excitatory and inhibitory postsynaptic potentials, the regional processing of the microcircuit surrounding the recording electrode, the cytoarchitecture of the recorded area, and the temporally synchronous fluctuations of the membrane potential in large neuronal groups (Buzsáki et al., 2012). Overall, however, LFPs and spike trains can be considered as the input and output stages of information processing, respectively.

In the present study, we tested the hypothesis that the processing of cognitive information associated with different aspects of a spatial categorization task involves different synchronous and lagged interactions of the inputs of a circuit, measured by LFPs.

## Materials and methods

### Animals

Two male rhesus monkeys (*Macaca mulatta*, 5 and 6 kg BW) were used in this study. The animals were on a regulated water schedule. Animal care conformed to the principles outlined in *the Guide for Care and Use of Laboratory Animals* (National Institutes for Health publication no. 85-23, revised 1985). The animal protocols were approved by the Institutional Review Board.

### Behavioral task

**Figure 1:**
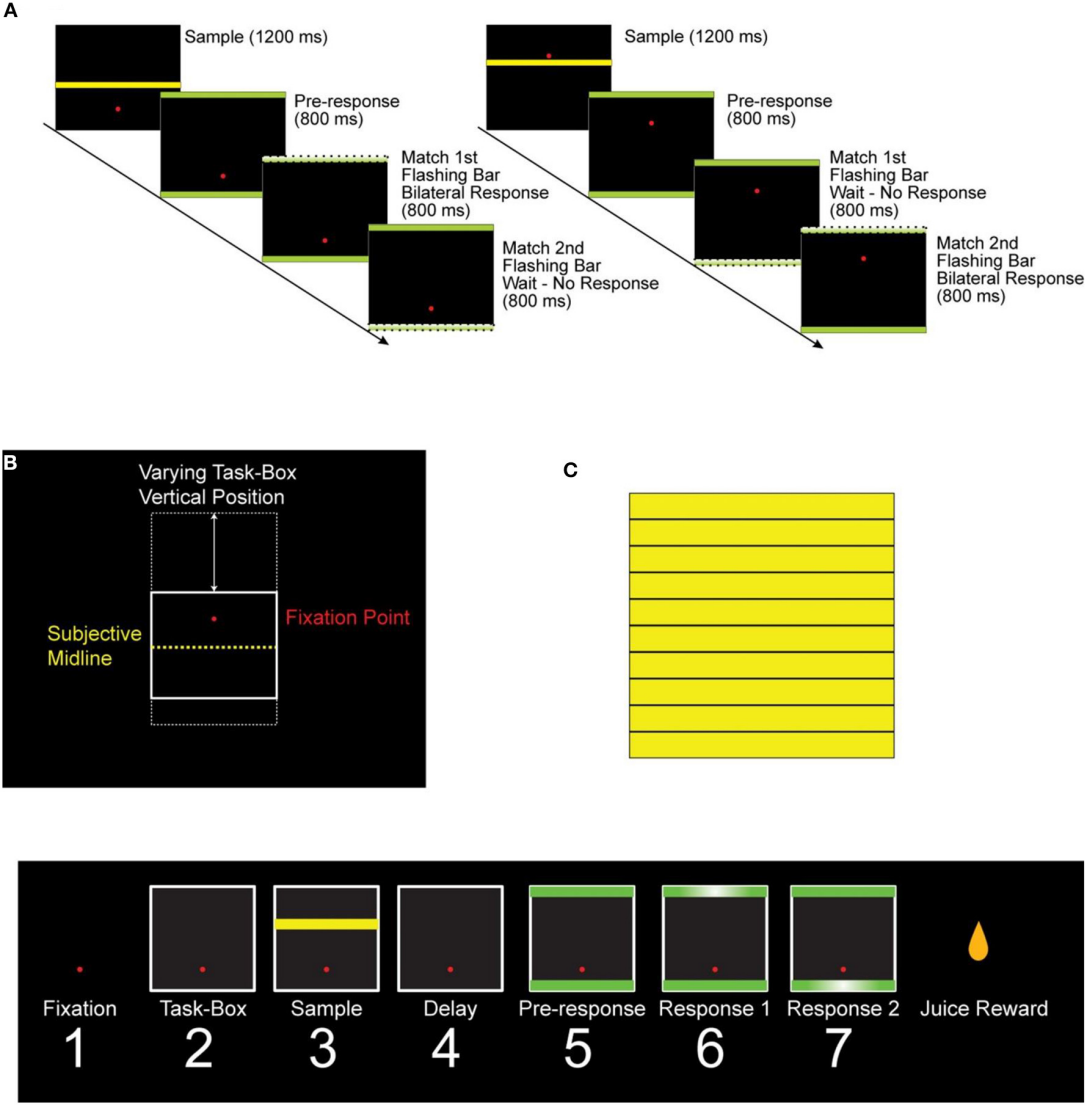
**“High/Low” delayed matched-to-sample space categorization task. (A)** Sample trials of the task. After the monkey fixated the red dot, a black task box appeared on a screen in front of the monkey. The vertical position of the box within the screen varied randomly in different trials **(B)**. Following a brief delay, a yellow sample bar was displayed for 1.2 s. Following another brief delay (0.5 s), two green response bars appeared simultaneously on the top and bottom of the task box. After a delay (1.5 s for monkey 1 and 0.8 s for monkey 2), the response bars would flash for 0.8 s in a random sequence, and the monkey had to match the sample bar to the hemi-space corresponding to the flashing bars by pressing simultaneously two foot pedals to receive a juice reward (1). S, sample period; PR, pre-response period; R1 and R2, first and second response periods, respectively. **(C)** The 10 intermediate heights that were used for the sample bar. A summary of the task epochs is shown in the bottom panel. Eye fixation was required throughout the duration of the trial. The monkeys performed very well in this task (75 and 90% correct for monkey 1 and 2, respectively).

We used a task (Figure 1) in which two monkeys categorized a sample stimulus to either one of two half-spaces (upper and lower) in a task box, as described in detail in Fortes et al. (2004). Since the task box changed location from trial to trial, the task involved categorization of a relative spatial cue. There were three periods of interest in the task (Figure 1). During the “sample period,” the monkey received all the information needed to categorize the stimulus bar; during the following “pre-response period” the monkey prepared the response which was elicited during one of the ensuing two “response periods.” From an information processing point of view, the sample period is the most demanding and crucial one, for it is during that period that the process of spatial categorization is taking place. Cognitive load decreases after that period and becomes smallest during the response time. To test the hypothesis that fine-grain (1 ms temporal resolution) synchronous and lagged neuronal interactions are involved in information processing, we recorded LFP activity from two cortical sites simultaneously in the posterior parietal cortex, based on the known involvement of area 7a in spatial cognitive processing.

### Neural recordings

**Figure 2:**
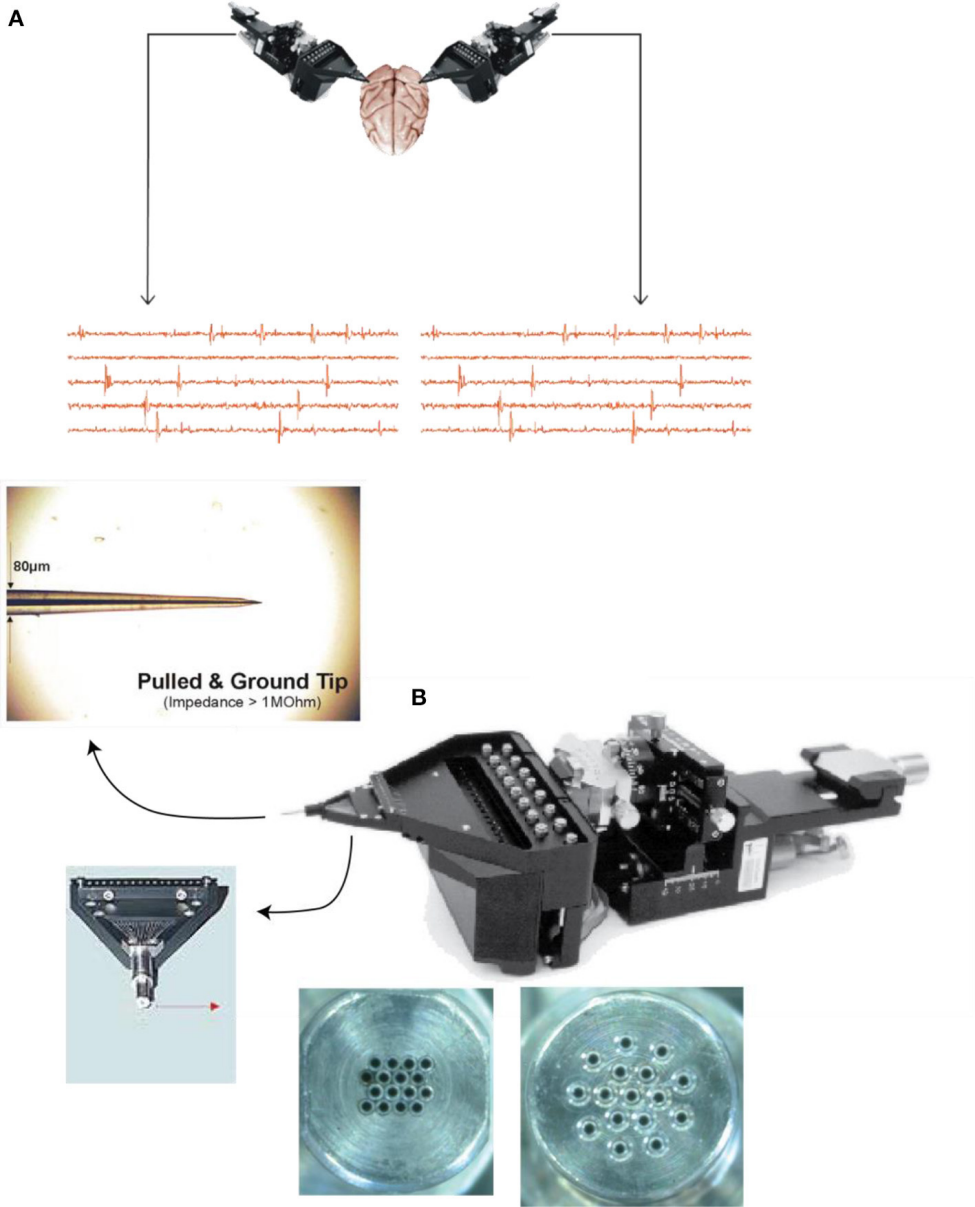
**Experimental setup illustrating (A) two 16-electrode Eckhorn matrices placed over symmetric sites in area 7a and representative multi-unit records, and (B) details of the recording system, a representative electrode tip, and two electrode head configurations**.

Neuronal activity was recorded extracellularly using two independently movable 16-microelectrode matrix systems (Eckhorn system, Thomas Recording, GMbH, Giessen, Germany, Figure 2B). The recording sites included symmetric areas 7a of the posterior parietal cortex, bilaterally (see Figure 2A). There were six recording sites in any given trial, of which three were in the left and three in the right hemisphere. The raw analog electrical activity was digitized at 40 kHz and stored in a personal computer via two PCI-DAS64/M2/16 high speed analog boards (Measurement Computing, Middleboro, MA). LFPs were extracted from three microelectrodes per matrix using a LFP-filter amplifier (Thomas Recording GmbH, Giessen, Germany) with a low-pass filter at 120 Hz and a high-pass filter at 0.1 Hz. All channels were recorded irrespective of neural activity during the task. The eye position was sampled at 200 Hz using an infrared video eye tracking system (ISCAN Inc., Burlington, MA). Two male rhesus macaque monkeys (weighing 8 and 7 kg) were used. They were prepared for recording using standard aseptic surgical techniques under Isoflurane (1–2%) gas anesthesia. In each animal, recording chambers (7 mm internal diameter) were implanted bilaterally, above a craniotomy overlying area 7a in each of the two hemispheres. Five titanium posts were attached to the skull with titanium screws, and a halo was attached to provide an anchor point to stabilize the head during neural recordings. Analgesia was provided for a period of several days following surgery (Buprenex, 0.05 mg/kg BID, i.m.).

### Data analysis

**Figure 3:**
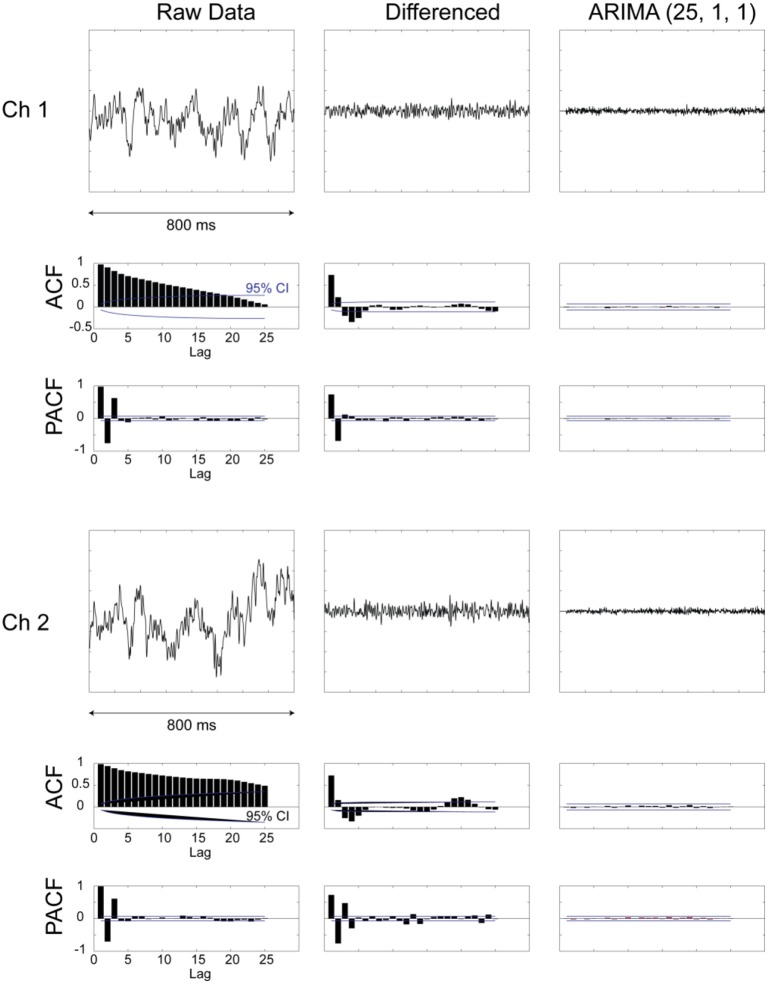
**Examples from two LPF recordings and their preprocessing for prewhitening**. Plots are shown for raw data, data after first-order differencing, and after applying an ARIMA [25,1,1] model. In addition, plots of autocorrelation function (ACF) and partial autocorrelation function (PACF) are shown for the different stages of data processing. It can be seen that the prewhitened data (right-most column) are devoid of any internal dependencies and evidenced by the flat ACF and PACF plots.

**Figure 4:**
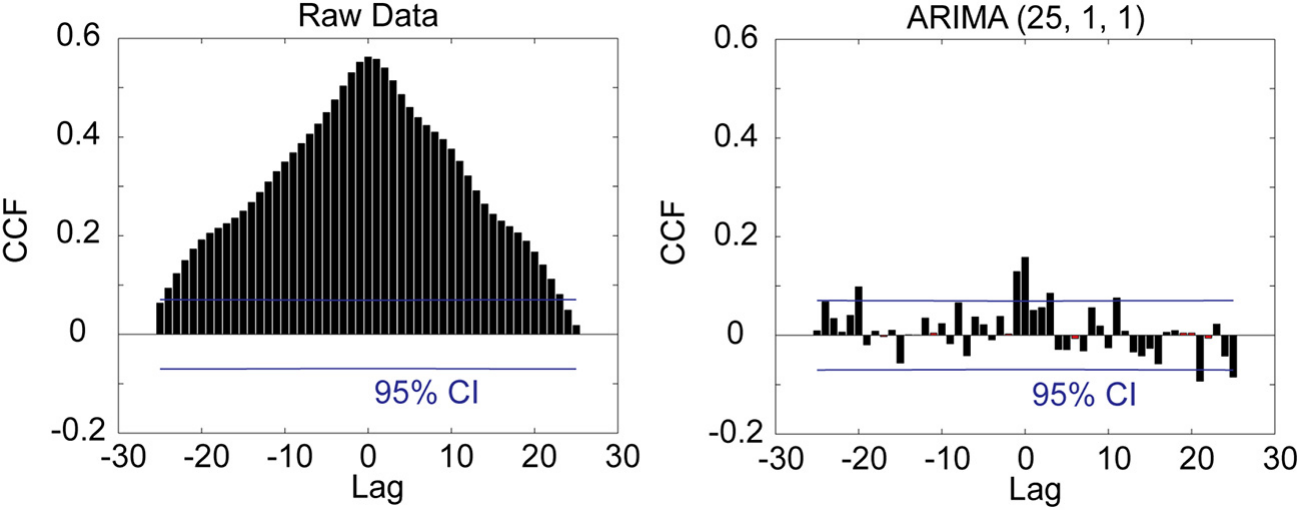
**Crosscorrelation (CCF) functions between the two LFP channels illustrated in Figure 3 for the raw data (left panel) and prewhitened data (right panel)**. The crosscorrelogram of the raw data is spurious.

The raw digitized extracellular record (collected at 40 kHz) was resampled (decimated) to 1 kHz, by picking every 40th sample of the original digitized data. The resulting time series were then prewhitened by taking the residuals after applying an [25,1,1] AutoRegressive Integrative Moving Average (ARIMA) model (Box and Jenkins, 1970; Priestley, 1981) (see Figure 3 for details). This model was arrived at after extensive model identification and yielded residuals (innovations) that were practically stationary with respect to the mean and variance, and flat autocorrelations (see Results below). The crosscorrelation function (±25 ms maximum lag) (Figure 4) was computed for all pairs of recorded LFP time series and for each one of the seven periods of the task (Figure 1). The following three measures were extracted from each crosscorrelogram: (a) The crosscorrelation at zero lag (*CC*^0^), indicating synchronicity, (b) the crosscorrelation with the maximum absolute value (*CC^max^*), and (c) the lag at which *CC^max^* occurred. For the purposes of this analysis, the sign of the lag was ignored, hence its absolute value was used (range: 0–25 ms); hereafter, we use “lag” to mean “absolute value of lag.” The signs of *CC*^0^ and *CC^max^* were retained and their statistical significance calculated using the assumption that the series are white noise. The crosscorrelations were z-transformed to normalize their distribution (Fisher, 1958):

(1) zCC=atanh(CC)

This is equivalent to

(2) $$zCC=\frac{\text{ln}\left(\frac{1+CC}{1-CC}\right)}{2}$$

Finally, standard statistical methods were performed to further analyze the data (Snedecor and Cochran, 1989), as mentioned below, using the IBM SPSS Statistics package, version 22.

## Results

### Synchronicity: *CC*^0^

#### General

There were 1,623,472 *CC*^0^ overall, from all trials and task periods (*N* = 883,291 within the same hemisphere and *N* = 740,181 across hemispheres). Of all *CC*^0^, 40.4% were statistically significant (*P* < 0.05). The percentage of statistically significant *CC*^0^ was 3.3× higher within the same hemisphere (59.3%) than across hemispheres (17.8%); these proportions differed significantly (*P* < 0.001) (Fliess, 1981). Finally, with respect to the sign of statistically significant *CC*^0^, the prevalence of positive *CC*^0^ was 1.24× higher (*P* < 0.001) within the same hemisphere (94.7%) than across hemispheres (75.8%); conversely, negative *CC*^0^ were more prevalent across hemispheres (24.2%) than within the same hemisphere (5.3%). These findings indicate that high resolution (1 ms) synchronicity is widely prevalent in the parietal cortex, and much more so within a hemisphere.

#### Modulation of *CC*^0^ by task period

**Figure 5:**
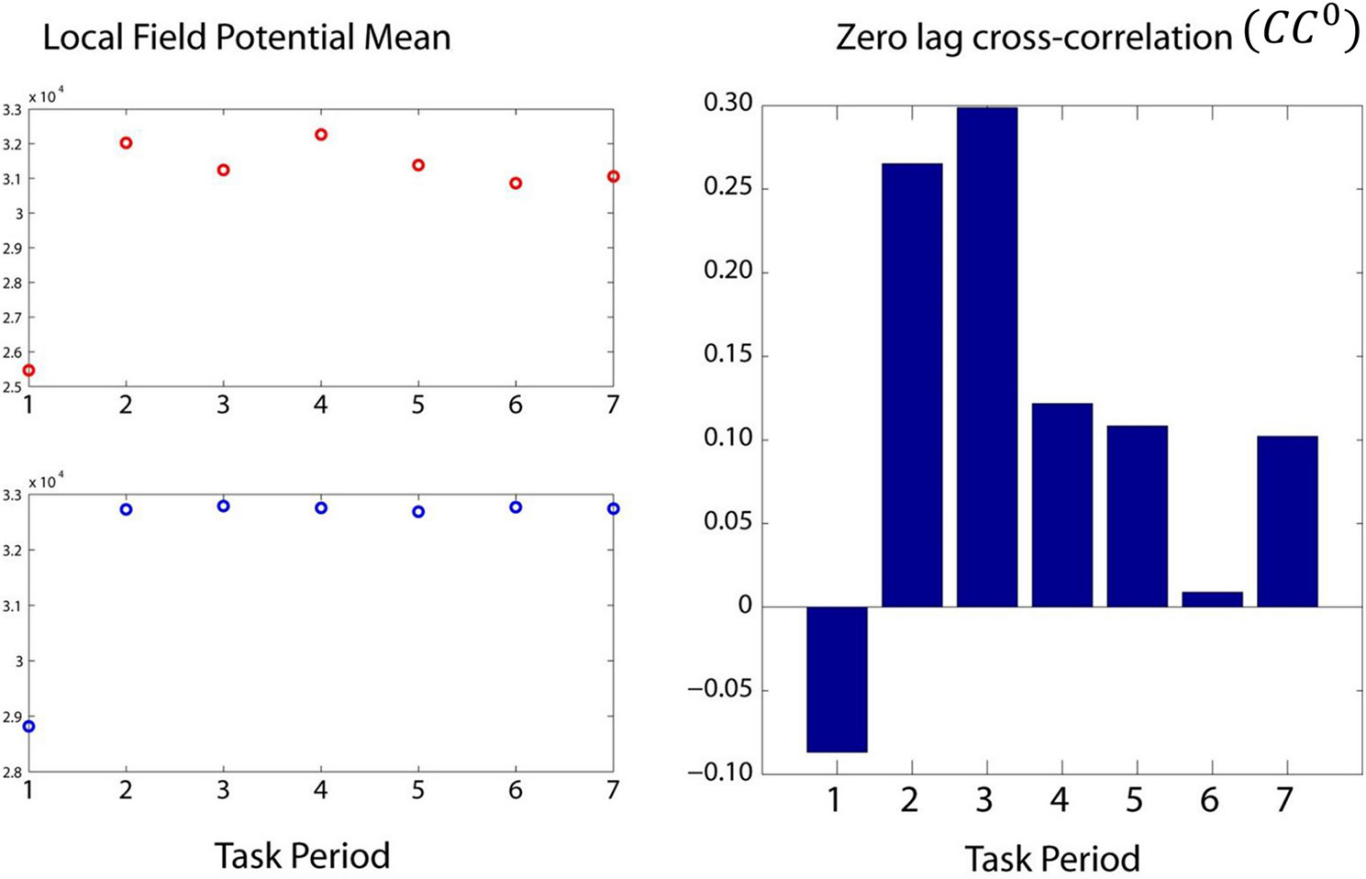
**Modulation of two LFPs (left panel) and their *zCC*^0^ (right panel) during the seven task periods**. Although the LFP magnitude increased after the first period, it did not show systematic modulation during the subsequent periods. In contrast, *zCC*^0^ was obviously modulated, peaking at period 3, the most cognitively demanding period.

**Figure 6:**
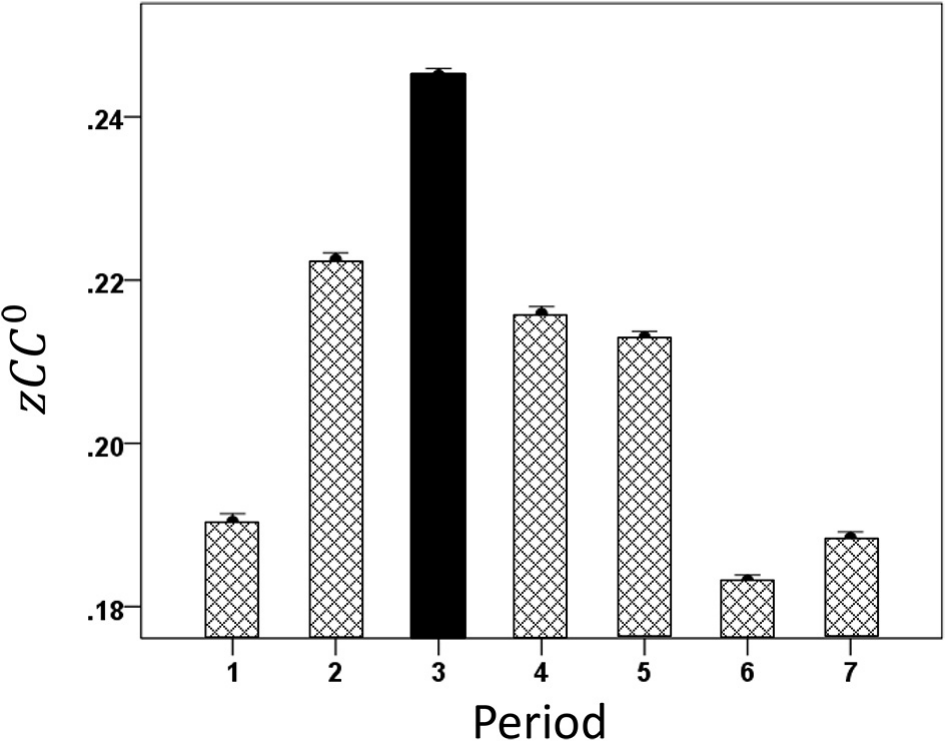
**Mean modulation (±s.e.m.) of same-hemisphere *zCC*^0^ during task periods, peaking at period 3 (see text for details)**.

**Figure 7:**
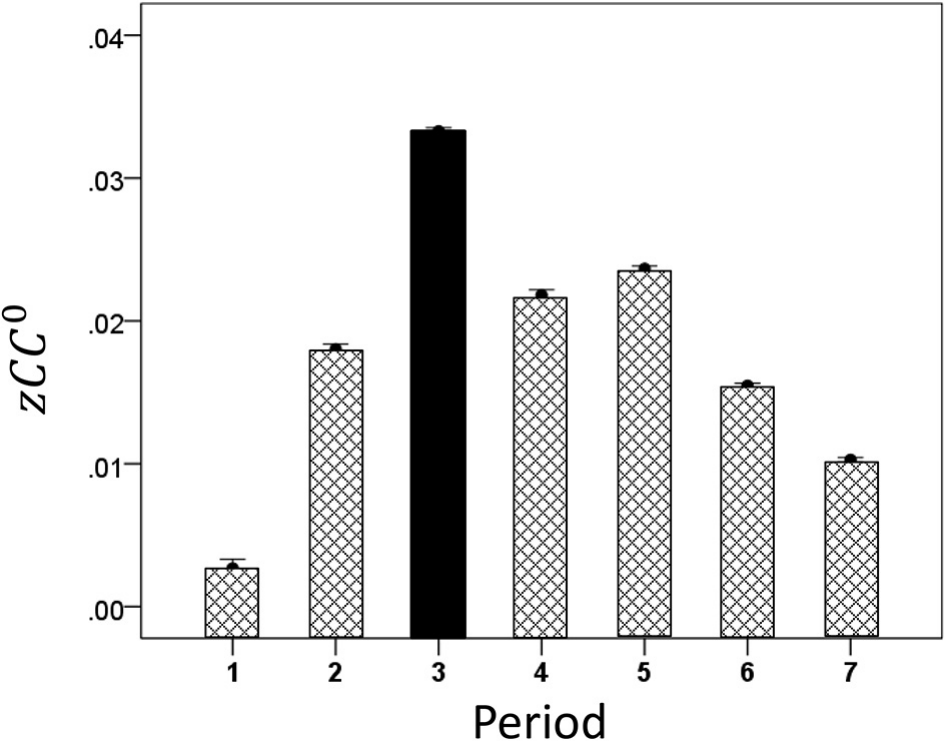
**Mean modulation (±s.e.m.) of opposite-hemispheres *zCC*^0^ during task periods, peaking at period 3 (see text for details)**.

By task design, the cognitive demand differed among periods, such that it was highest during the sample period (when the test bar had to be categorized as High or Low, Figure 1) and lowest during the initial fixation period and the last two response periods. We found that *CC*^0^ was modulated across task periods, being highest in the sample period and much lower at the beginning and the end of the trial. An example from a LFP pair is illustrated in Figure 5. Mean values across all trials and periods are shown in Figures 6, 7 for recordings in the same and opposite hemispheres, respectively. We evaluated the statistical significance of this variation by performing a repeated measures analysis of covariance (ANCOVA) in which the seven periods of the task were the repeated measures factor, the hemisphere (same or across) and repetition were “between-subjects” factors, and the vertical position of the task box on the screen and the elapsed time for recording the trial were covariates. Bonferroni tests in the ANCOVA showed that *zCC*^0^ in the sample period differed highly significantly from all others (*P* < 0.001) both within the same hemisphere and across hemispheres.

### Lagged neural interactions: *CC^max^*

#### General

**Figure 8:**
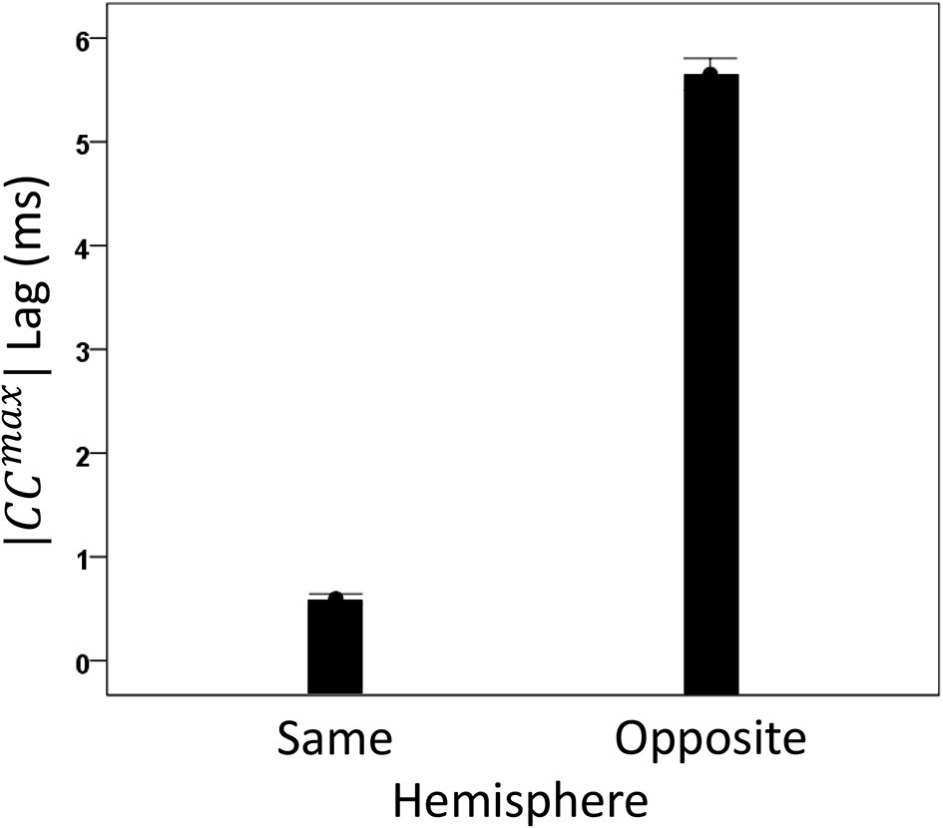
**Mean lags (±s.e.m.) of |*CC^max^*| for same- and opposite hemisphere recordings (see text for details)**.

**Figure 9:**
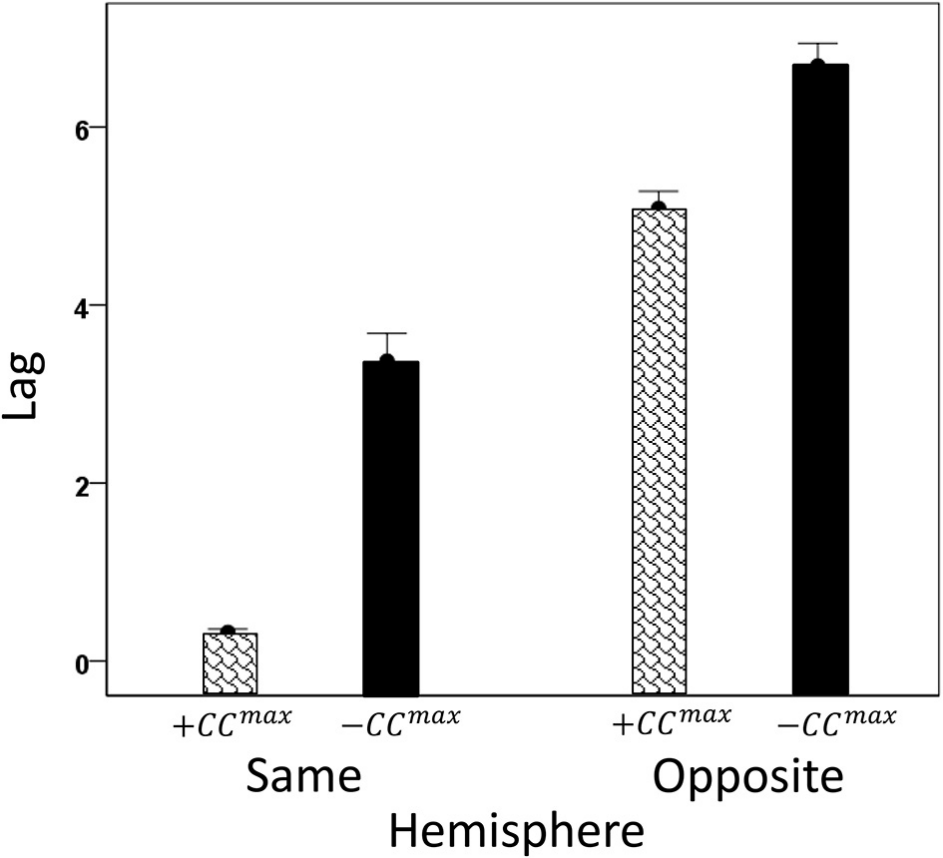
**Mean *CC^max^* lags (±s.e.m.) for different recording sites and *CC^max^* sign (see text for details)**.

**Figure 10:**
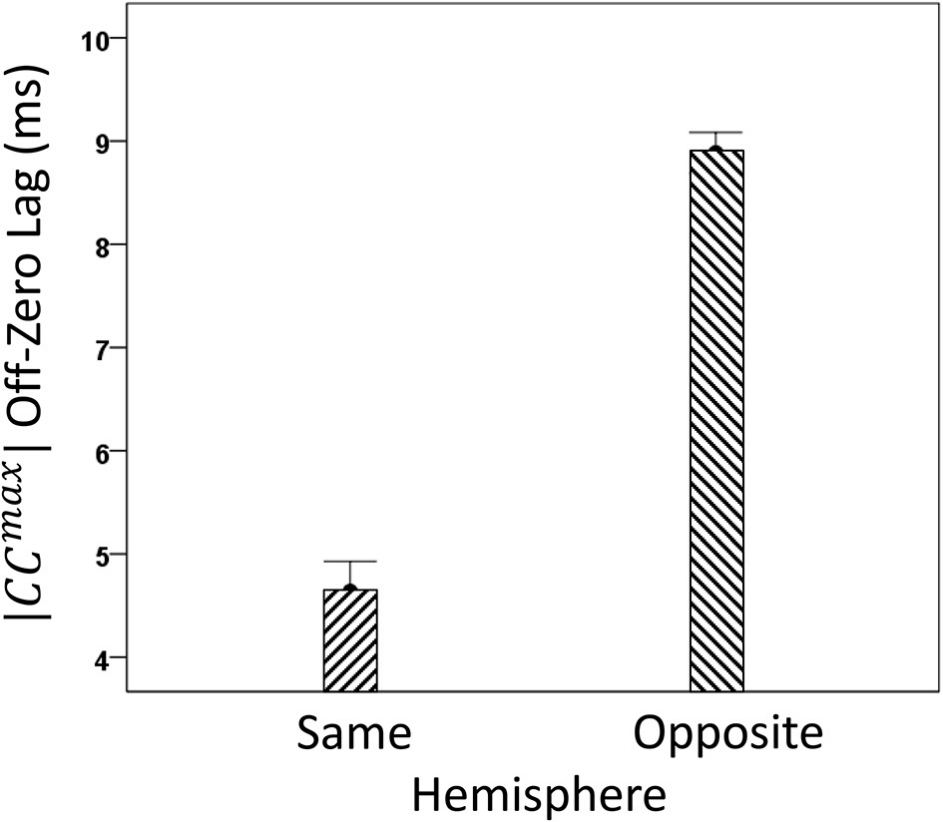
**Mean off-zero lags (±s.e.m.) of |*CC^max^*| for same- and opposite hemisphere recordings (see text for details)**.

**Figure 11:**
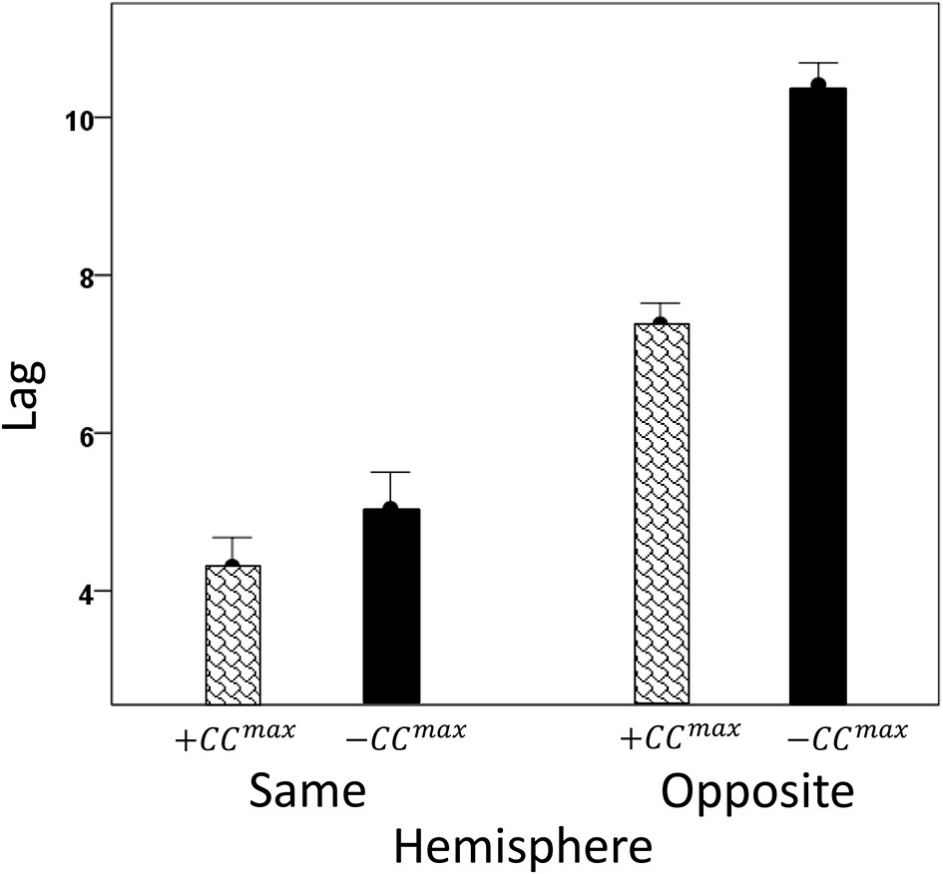
**Mean off-zero *CC^max^* lags (±s.e.m.) for different recording sites and *CC^max^* sign (see text for details)**.

We analyzed *CC^max^* and corresponding lags for periods 3, 5, 6, and 7 in 4824 trials with correct behavioral outcomes; 2556 were from LFP pairs within the same hemisphere, and 2268 were from opposite hemispheres. We found the following. (a) *CC^max^* occurred at zero lag in 87.2% of LFP pairs recorded in the same hemisphere and only in 36.4% (244/567) of recordings between opposite hemispheres; these two proportions differed highly significantly (*P* < 0.001). Conversely, only 12.8% *CC^max^* occurred at off-zero lags in same-hemisphere recordings, as compared to 63.6% in opposite-hemisphere recordings. (b) The magnitude of |*CC^max^*| varied inversely with lag, such that the longer the lag the weaker the correlation. This effect was much stronger for opposite-hemisphere than same-hemisphere recordings: the correlation coefficient between |*CC^max^*| and absolute lag was −0.469 for the former, and −0.160 for the latter (*P* < 0.001 for both). (c) Lags were appreciably longer for recordings from opposite hemispheres (mean ± s.e.m., 5.65 ± 0.154) than from the same hemisphere (0.432 ± 0.048) (Figure 8). In addition, lags for negative *CC^max^* were consistently longer than for positive *CC^max^* (Figure 9). An ANOVA showed that both main effects of recording sites (same/opposite hemisphere) and *CC^max^* sign (positive/negative) were highly significant (*P* < 0.001 for each, *F*-test) as was their interaction too (*P* = 0.001, *F*-test). This significant interaction reflects the steeper increase in lag (from +*CC^max^* to −*CC^max^*) in the same- than the opposite-hemisphere recordings (Figure 9). (d) Given that a large proportion of lags were at zero, we carried out an additional analysis of off-zero lags, after excluding zero lags. The results are shown in Figures 10, 11. It can be seen that the effect of recording site and *CC^max^* sign were in the same direction as when lags at zero were included (Figures 8, 9). In addition, an ANOVA on the off-zero lag data showed that both main effects of recording sites (same/opposite hemisphere) and *CC^max^* sign (positive/negative) were highly significant (*P* < 0.001 for each, *F*-test) as was their interaction too (*P* = 0.008, *F*-test).

#### Modulation of *CC^max^* and lag by task period

**Figure 12:**
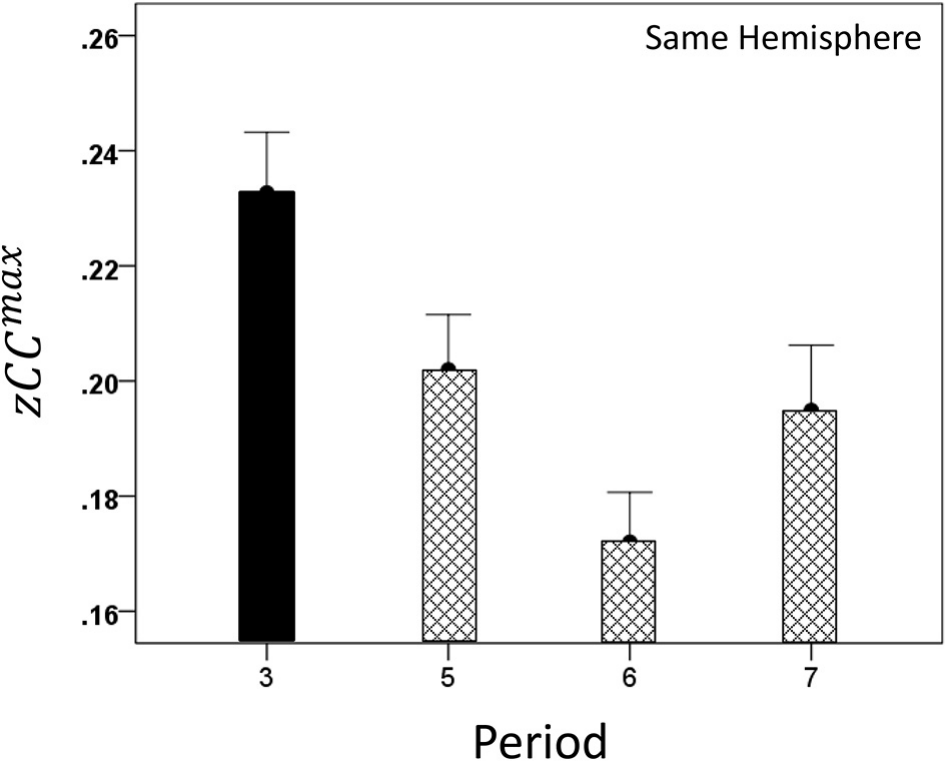
**Modulation of same-hemisphere *zCC*^max^ during task periods, peaking at period 3 (see text for details)**.

**Figure 13:**
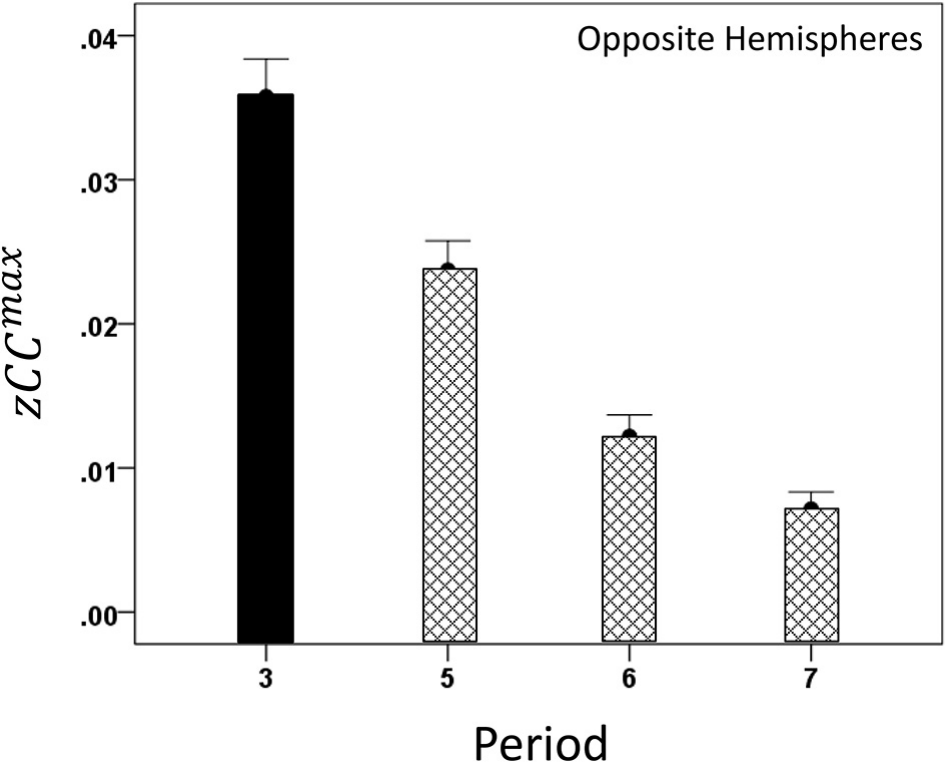
**Modulation of opposite-hemisphere *zCC*^max^ during task periods, peaking at period 3 (see text for details)**.

**Figure 14:**
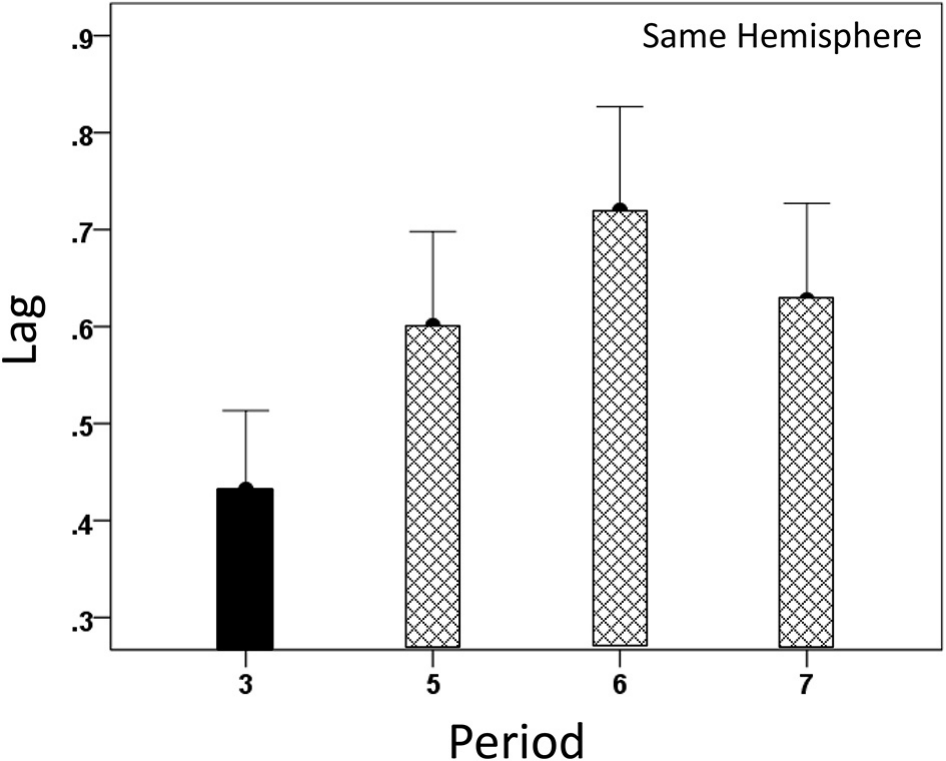
**Modulation of same-hemisphere *CC^max^* lag during task periods, being shortest at period 3 (see text for details)**.

**Figure 15:**
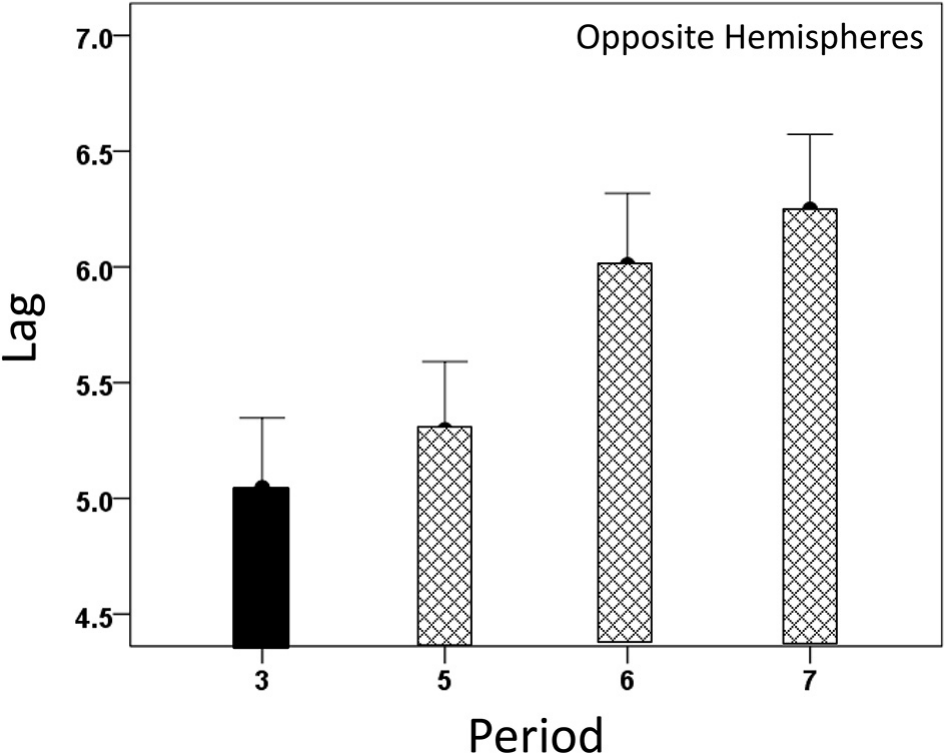
**Modulation of opposite-hemisphere *CC^max^* lag during task periods, being shortest at period 3 (see text for details)**.

**Figure 16:**
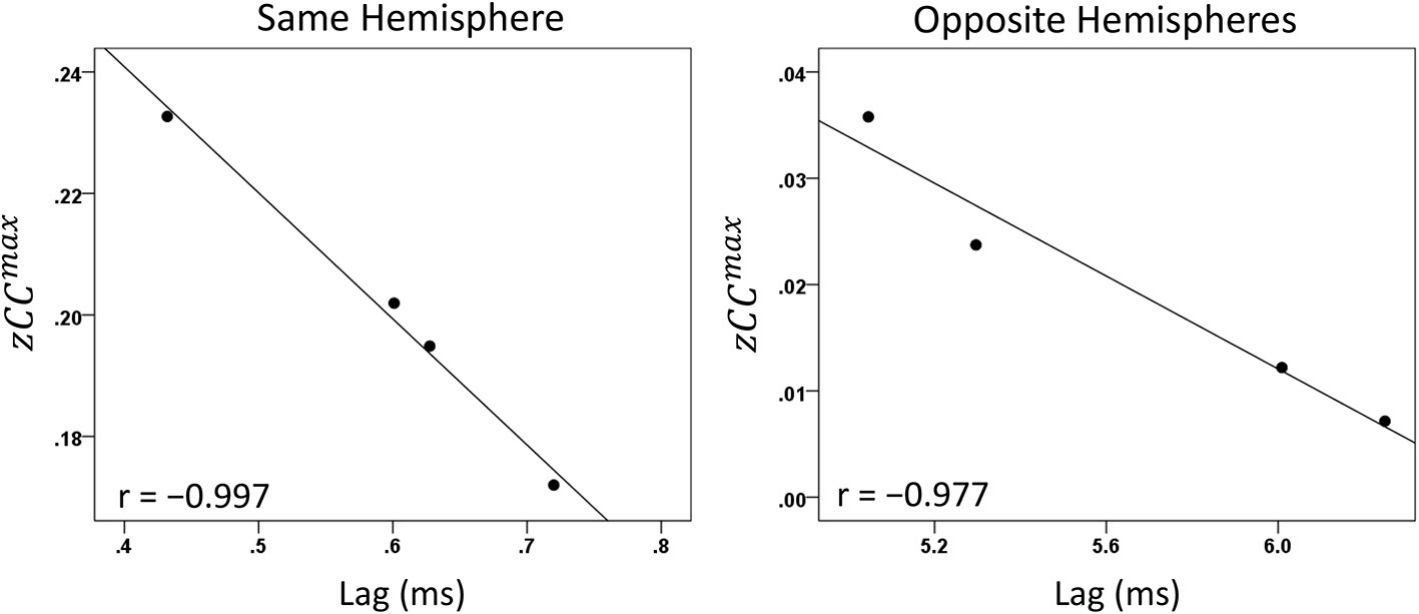
**Mean *CC^max^* per task period is plotted against its mean lag for same- and opposite-hemisphere recordings (see text for details)**.

Both *CC^max^* and its lag were modulated systematically by cognitive load, as it varied among task periods. *CC^max^* was highest during the sample period for both same- (Figure 12) and opposite-hemisphere (Figure 13) recordings. In contrast, lag was shortest during the sample period for both recording sites (Figures 14, 15). ANOVAs revealed highly statistically effects of the period and recording site on *CC^max^* (*P* < 0.001 for both, *F*-test) and on lag (*P* = 0.003 for period and *P* < 0.001 for recording site, *F*-test). Period × Recording Site interactions were not statistically significant for either *CC^max^* (*P* = 0.094) or lag (*P* = 0.06). Finally, *CC^max^* varied inversely with lag across the four periods tested. Figure 16 plots the mean *CC^max^* against the mean lag for the two recording sites; the high negative correlations shown were statistically significant (*P* = 0.005 and 0.023 for same and opposite hemispheres, respectively).

## Discussion

LFP activity has been recently implicated in several studies (Hwang and Andersen, 2010; Kelly et al., 2010; Purcell et al., 2012; Maris et al., 2013; Bartolo et al., 2014). For example, in the parietal cortex, LFPs can predict behavioral states associated with saccade and reaching movements (Scherberger et al., 2005), and in the middle temporal area, LFPs are correlated with processing of motion direction and perceptual judgments of speed (Liu and Newsome, 2006). In addition, LFP activity has been used in coding hand movement target and velocity (Mehring et al., 2003), discrimination between preferred and anti-preferred direction and predicting the time of a planned movement (Pesaran et al., 2002), perceptual suppression (Wilke et al., 2006), and selection of image category in the human medial temporal lobe (Kraskov et al., 2007). Since LFPs reflect integrated synaptic potentials, their intensity reflects variation at the input stage of local processing, whereas action potentials represent the outputs (Mitzdorf, 1987). Interestingly, interactions among these output spike trains, including synchronicity, seem to contain very little additional information that is not readily available in the discharge rate of individual neurons (Zohary et al., 1994; Averbeck et al., 2006). In contrast, the processing of neuronal information in the time scale of milliseconds, as demonstrated in the present study, should occur at the level of the input and subsequent stages prior to the output of the network. These findings are in general accord with those of an earlier study which showed that spike-triggered LFPs were modulated by selective attention (Fries et al., 2001). Thus, the issue of information carried by synchronicity is refocused away from the spike output (Zohary et al., 1994; Averbeck et al., 2006) on to the synaptic inputs. An understanding of this input modulation could shed light into the neural processing of selectivity, saliency and other aspects of cognitive function.

### Synchronous (zero-lag) interactions

With respect to zero-lag interactions, there are two plausible sources of synchronized inputs to a cortical area, namely recurrent collaterals of pyramidal cell axons (Stefanis and Jasper, 1964a,b; Brooks and Asanuma, 1965) and thalamocortical inputs (Jones, 2001; Bruno and Sakmann, 2006). The former, and thalamic inputs directed specifically to a given area arising from parvalbumin-immunoreactive neurons (Jones, 2001), could account for local (within-area) input synchronization, whereas widely and multifocally distributed thalamic inputs, arising from calbindin-immunoreactive neurons (Jones, 2001), could subserve long-distance (between-areas) input synchronization. The results of the present study demonstrated that that such inputs can be finely synchronized (at 1 ms temporal resolution). This synchronization was mostly positive and occurred more frequently within area 7a of the same hemisphere, whereas it was mostly negative and occurred less frequently across symmetric 7a sites in the two hemispheres. Nevertheless, in spite of these differences, the strength of LFP synchronization was modulated in a similar fashion by the cognitive load of the task. These findings reinforce the role of area 7a in spatial cognitive processing, as shown in different studies (Merchant et al., 2001, 2003, 2004a,b, 2005), and extend the neuronal mechanisms involved from single cell activity (Chafee et al., 2005; Crowe et al., 2005, 2013; Chafee and Crowe, 2012) to encompass LFP synchronicity.

### Lagged interactions

Our results demonstrated the orderly modulation of lagged neural interactions by cognitive load. Simply, interactions became stronger and more synchronous (at shorter lag) during the sample period, when the bar to be categorized was presented. This effect was observed both for interactions within as well as across hemispheres, even though the magnitude of correlations and lags were much smaller in the latter than in the former case. Altogether, our findings establish the strength and lag of neural interactions as meaningful variables under cognitive control.

Finally, our results provide a novel source of correspondence between neurophysiological and neuroanatomical measurement, namely on callosal conduction delays. Specifically, detailed measurements of white matter variables and fiber characteristics have furnished estimates of callosal conduction delays in monkeys and humans (Caminiti et al., 2013). For the monkey, evidence based on fiber histology provided an estimated mean callosal conduction delay between posterior parietal cortices of 6.42 ± 3.11 ms (mean ± *SD*) (Caminiti et al., 2013, Table 1) which is statistically indistinguishable from our estimate of 5.65 ± 7.34 ms (Figure 8). To our knowledge, this is the first time that crosscorrelation of LFP time series has been used to yield such an accurate estimate of callosal conduction delays in such a close agreement to estimates derived from purely anatomical measurements. We believe that this was made possible by the proper prewhitening procedure which converted the LFP time series to white noise, thus allowing for a correct estimation of neuronal interactions.

### Conflict of interest statement

The authors declare that the research was conducted in the absence of any commercial or financial relationships that could be construed as a potential conflict of interest.
